# Seeds used for Bodhi beads in China

**DOI:** 10.1186/1746-4269-10-15

**Published:** 2014-01-30

**Authors:** Feifei Li, Jianqin Li, Bo Liu, Jingxian Zhuo, Chunlin Long

**Affiliations:** 1 College of Life and Environmental Sciences, Minzu University of China, Beijing 100081, PR China; 2 Faculty of Forestry, Southwest Forestry University, Kunming 650224, PR China; 3 Kunming Institute of Botany, Chinese Academy of Sciences, Kunming 650201, PR China; 4 College of Agronomy and Biotechnology, Yunnan Agricultural University, Kunming 650201, PR China

**Keywords:** Bodhi beads, Seeds, Buddhist culture

## Abstract

**Background:**

Bodhi beads are a Buddhist prayer item made from seeds. Bodhi beads have a large and emerging market in China, and demand for the beads has particularly increased in Buddhism regions, especially Tibet. Many people have started to focus on and collect Bodhi beads and to develop a Bodhi bead culture. But no research has examined the source plants of Bodhi beads. Therefore, ethnobotanical surveys were conducted in six provinces of China to investigate and document Bodhi bead plants. Reasons for the development of Bodhi bead culture were also discussed.

**Methods:**

Six provinces of China were selected for market surveys. Information was collected using semi-structured interviews, key informant interviews, and participatory observation with traders, tourists, and local residents. Barkhor Street in Lhasa was focused on during market surveys because it is one of the most popular streets in China.

**Results:**

Forty-seven species (including 2 varieties) in 19 families and 39 genera represented 52 types of Bodhi beads that were collected. The most popular Bodhi bead plants have a long history and religious significance. Most Bodhi bead plants can be used as medicine or food, and their seeds or fruits are the main elements in these uses. ‘Bodhi seeds’ have been historically used in other countries for making ornaments, especially seeds of the legume family. Many factors helped form Bodhi bead culture in China, but its foundation was in Indian Buddhist culture.

**Conclusions:**

As one of the earliest adornment materials, seeds played an important role for human production and life. Complex sources of Bodhi beads have different cultural and historical significance. People bought and collected Bodhi beads to reflect their love and admiration for the plants. Thus, the documentation of Bodhi bead plants can serve as a basis for future investigation of Bodhi bead culture and modern Buddhist culture.

## Background

Humans use seeds (include the fruits with pericarps cannot be easily removed, commonly called ‘seeds’) in many ways. Seeds are colorful, durable, and easy to access, so humans have drilled and strung seeds into necklaces and bracelets for thousands of years. The oldest seed ornaments were discovered in Africa and can be dated to the Middle Stone Age (280,000 to 45,000 years ago) [1]. Moreover, due to the variety of medicinal and edible properties of plants and trees, people placed high value on this type of vegetation, and this feeling has continued to modern times [2]. Some people endow beads with specific meaning for averting disaster, developing wisdom, and soothing and relaxing.

Bodhi beads are Buddhist prayer items that have been traditional tools for counting while reciting a mantra, as prayer beads have been used in other world religions. The Bodhi beads are called *Pu Ti Zi* in Chinese: *Pu Ti* means Bodhi tree (*Ficus religiosa*), and *Zi* means seed; but Bodhi beads are not made of the seeds of a Bodhi tree (*Ficus religiosa*). The name *Pu Ti Zi* first appeared in the early medical text, ‘*Compendium of Materia Medica*’, which referred to the seeds of *Coix lacryma-jobi*[3]. In addition, seeds of *Sapindus delavayi* and *Sapindus tomentosus* were called *Pu Ti Zi* or ‘Bodhi seeds’ in Yunnan [4]. In modern times, ‘Bodhi seeds’ do not refer to any particular plant but instead refer generally to seeds and the fruits of various plants used to make prayer beads.

Bodhi beads have an important position in Tibetan Buddhism and are very popular in Tibet (the Xizang Tibetan Autonomous Region). The culture of Bodhi beads spread throughout Tibet and other provinces of China, and there are dozens to hundreds of Bodhi beads with different meanings. Recently, people have begun to wear Bodhi beads as a kind of praying or blessing ornament, in addition to functioning as a prayer bead. The Bodhi bead culture has developed in China in the form of cultural supplies, such as writing brushes and ink sticks. Several ‘Bodhi seeds’ of similar size, shape, and pattern are strung into strings of prayer beads. People frequently touch the beads with their hands to make the beads luster like jade due to secretion from human skin. Moreover, the beads are carefully modified to protect from scarring in inappropriate temperature and humidity surroundings.

Ethnobotanists study how plants are used for food and medicine but are also interested in plant adornments because non-mainstream use of plants can reflect relationships between cultures and (uses of) plants in other aspects. Surveys such as that conducted by Armstrong have shown that many botanical jewelries in different places were made by seeds and indicated the high value placed on the seeds [5,6]. Over 165 plant species used for human adornment in India were identified and listed [7].

Recently, there has been increasing interest in the commercial value of Bodhi beads, and many books about the culture of Bodhi beads have appeared in China. Although the books and related materials focus on the meaning of each kind of Bodhi bead, almost all publications ignore the question of which plant or plants produced the Bodhi seeds. The beads have been commonly described as ‘seeds of rare plants’, and this description has fostered a belief that the value of Bodhi beads is related to the scarcity of their sources and not to their aesthetic and cultural value. To help correct this misunderstanding, we investigated the plant sources of ‘Bodhi seeds’ or *Pu Ti Zi* and explored the culture values of Bodhi bead plants.

## Methods

### Study area

Surveys were conducted in local markets and e-commercial platforms of six provinces in China: Tibet, Yunnan, Fujian, Zhejiang, Beijing, and Guangdong. Field work was conducted from September to October 2012 and August to September 2013. Barkhor Street (29°39′ N, 91°7′ E) is a famous street circling Jokhang Temple in Lhasa, Tibet, and was the main local market surveyed. The traditional culture and local lifestyle have been well-preserved for more than 1300 years. We choose this market because of the wide variety of Bodhi beads traded there between vendors, pilgrims, and tourists from Nepal, Bhutan, India, and other places in China. It is the most important commercial center, folk-custom heritage, tourist attraction [8], and shopping center in Lhasa. Other Bodhi bead trading centers were also investigated, including Shangri-la and Kunming in Yunnan, Xiamen in Fujian, and cultural markets and temples in Beijing.

### Data collection

Bodhi beads samples were purchased from Barkhor Street markets and e-commercial platforms. A detailed inventory and related information for the Bodhi beads were prepared from semi-structured interviews, key informant interviews, and participatory observation with traders, tourists, and local residents. In order to access the popularity of Bodhi beads in Chinese markets, various Bodhi beads in 126 shops of six provinces were studied. The number of shops that each Bodhi bead was sold in was counted for an occurrence frequency (OF) calculation; for example, if N shops sold Bodhi bead type ‘A’ (N ≥ 1), then the occurrence frequency of ‘A’ is OF(A) = N/126 × 100%.

### Taxonomic identification

All seeds were identified according to morphological characters and geographical origins using standard literatures [9-12] and cross-referenced with herbarium specimens deposited at PE (Herbarium, Institute of Botany, Chinese Academy of Sciences). A literature review was used to record additional information, including whether the seeds were used as human food, landscape plants, medicine, condiments, or building timber in China and whether the seeds are used for ornaments in other countries [2,6,7,13-24].

## Results and discussion

### Bodhi bead plant diversity and distribution

Fifty-two types of Bodhi beads were documented (Table 1), made of seeds from 47 species (including 2 varieties) belonging to 19 families and 39 genera. Among them were only one endangered (EN) species (*Latania loddigesii* – ‘Xian-Zhi’) in IUCN [25] and two critically endangered (CR) species (*Dracontomelon macrocarpum* – ‘Grimace’ and *Cycas revoluta* – ‘Buddha-mind’) in the China Species Red List [26]. The majority of Bodhi seed plants belonged to Arecaceae (12 genera and 13 identified species) and Fabaceae (7 genera and 8 identified species). The genera represented by the highest number of species was *Caesalpinia* (3 identified species). Most seeds of a particular species can produce only one type of Bodhi bead. However, different degrees of processing of *Corypha umbraculifera*, *Daemonorops jenkinsiana*, and *Elaeis guineensis* can be used to make different types of Bodhi bead. The seeds with 2-, 3- or 4-loculed drupes of *Ziziphus abyssinica* can be used to make ‘Phoenix eye’, ‘Dragon eye’, and ‘Kylin eye’, respectively. Chinese names of most Bodhi beads are according to the morphological characteristics of their respective seeds; for example, the beads called ‘Moon and Stars’ usually have an ivory surface with small holes (moons) and tiny black dots (stars). Seeds of *Caesalpinia bonduc* are grayish, shiny, ovoid to globose, and look like the moon; therefore, the name of the corresponding Bodhi bead is ‘Moon seed’.

**Table 1:** Plants used for making Bodhi beads

**No.**	**Chinese name**	**Common name**	**Family name**	**Scientific name**	**Occurrence frequency**	**Additional use(s) in China**	**Other countries**	**Distribution**
1	Wu yan liu tong 五眼六通	Five eyes six sense	Anacardiaceae	*Choerospondias axillaris* (Roxb.) B.L. Burtt & A.W. Hill	26.19	Medicinal, edible, timber plant		*
2	Gui lian 鬼脸	Grimace	Anacardiaceae	*Dracontomelon macrocarpum* H.L. Li	18.25	Medicinal, edible, landscape plant		*
3	Xue lian zi 雪莲子	Snow lotus seed	Anacardiaceae	*Pistacia vera* L.	14.29	Medicinal, edible plant		**
4	Jin yuan bao 金元宝	Gold ingot	Apocynaceae	*Thevetia peruviana* (Persoon) K. Schumann	7.14	Medicinal, landscape plant	India [7], Amazonian tribes [13]	*
5	Zi guang feng yan 紫光凤眼	Purple phoenix eye	Arecaceae	*Archontophoenix alexandrae* (F. Muell.) H. Wendl. & Drude	0.79	andscape plant		**
6	Jin si bao ta 金丝宝塔	Golden pagoda	Arecaceae	*Areca catechu* L.	15.08	Medicinal, edible plant	India [7]	*
7	Yu mi 玉米	Corn	Arecaceae	*Areca triandra* Roxb. ex Buch.-Ham.	6.35	landscape, fiber plant		**
8	Jin xian 金线	Gold thread	Arecaceae	*Caryota maxima* Blume ex Mart.	38.89	Landscape, edible plant		*
9	Pu ti gen 菩提根	Bodhi root	Arecaceae	*Corypha umbraculifera* L.	58.73	Landscape, edible, fiber plant	India [7,14]	**
10	Hu ban 虎斑	Tiger brindle	Arecaceae	*Corypha umbraculifera* L.	19.84	Landscape, edible, fiber plant	India	**
11	Mi gua 蜜瓜	Honeydew melon	Arecaceae	*Corypha umbraculifera* L.	13.49	Landscape, edible, fiber plant	India	**
12	Xing yue 星月	Moon and star	Arecaceae	*Daemonorops jenkinsiana* (Griff.) Mart.	88.10	Medicinal, landscape, fiber plant		*
13	Jin chan 金蝉	Golden cicada	Arecaceae	*Daemonorops jenkinsiana* (Griff.) Mart.	9.52	Medicinal , landscape, fiber plant		*
14	Mo ni zi 摩尼子	Mani	Arecaceae	*Daemonorops jenkinsiana* (Griff.) Mart.	8.73	Medicinal, landscape, fiber plant		*
15	Bai yu 白玉	White jade	Arecaceae	*Elaeis guineensis* Jacq.	15.08	Medicinal, oil-bearing plant		**
16	San yan jin zhu 三眼金猪	Three eyes gloden pig	Arecaceae	*Elaeis guineensis* Jacq.	13.49	Landscape, oil-bearing, fiber plant		**
17	Xian zhi 仙枝	Xian-Zhi	Arecaceae	*Latania loddigesii* Mart.	0.79	Landscape plant		**
18	Xiang ya guo 象牙果	Ivory nut	Arecaceae	*Phytelephas macrocarpa* Ruiz & Pav.	15.08	-	South America [15]	***
19	Zhao cai shu 招财鼠	Money rat	Arecaceae	*Syagrus romanzoffiana* (Cham.) Glassman	24.60	Landscape plant		**
20	Jin fo zhu 金佛珠	Gold Bead	Arecaceae	*Trachycarpus fortunei* (Hook.) H. Wendl.	5.56	Medicinal, landscape, fiber plant		**
21	Fo yan 佛眼	Buddha-eye	Arecaceae	*Washingtonia filifera* (Linden ex André) H. Wendl. ex de Bary	0.79	Landscape plant		**
22	Qian si 千丝	Countless ties	Arecaceae	*Wodyetia bifurcata* A.K. Irvine	3.97	Landscape		**
23	Gan lan 橄榄	Chinese white olive	Burseraceae	*Canarium pimela* K.D. Koenig	13.49	Medicinal, edible plant		*
24	Fo xin 佛心	Buddha-mind	Cycadaceae	*Cycas revoluta* Thunb.	1.59	Medicinal, landscape plant		*
25	Jin gang 金刚	King Kong	Elaeocarpaceae	*Elaeocarpus angustifolius* Blume	92.86	Medicinal, landscape, edible plant	India, Nepal [2]	*
		King Kong (2- furrows)	Elaeocarpaceae	*Elaeocarpus hainanensis* Oliv.	12.70	Medicinal, landscape, edible plant		*
26	A xiu luo 阿修罗	Asura	Euphorbiaceae	*Aleurites moluccana* (L.) Willd.	7.14	Medicinal, landscape, oil-bearing plant	USA (Hawaii), French Polynesia (Tahiti) [6,16]	*
27	Fu gui zi 富贵子	Riches and honour seed	Fabaceae	*Gleditsia sinensis* Lam.	11.11	Medicinal, timber, oil-bearing plant		*
28	Tian zhu 天竺	Tenjiku	Fagaceae	*Lithocarpus corneus* (Lour.) Rehder	14.29	Timber plant		*
29	Yi yi 薏苡	Job’s tears	Gramineae	*Coix lacryma-jobi* L.	2.38	Medicinal, edible plant	India [7,18,20], Colombia [17]	*
30	He tao 核桃	Playing Walnut	Juglandaceae	*Juglans regia* L.	10.32	Medicinal, edible, timber plant		*
31	Xiang si zi 相思子	Lovesick	Fabaceae	*Abrus precatorius* L.	3.97	Medicinal plant	India [7], Europe, and America [21]	*
32	Li yu lin 鲤鱼鳞	Carp’s scales	Fabaceae	*Acacia confusa* Merr.	2.38	Medicinal plant		*
33	Hong xin 红心	Red heart	Fabaceae	*Adenanthera pavonina* L.	38.89	Medicinal plant	India [7], Amazonia [22]	*
34	Mian qie 缅茄	Doussie	Fabaceae	*Afzelia xylocarpa* (Kurz) Craib	30.16	Medicinal plant		**
35	Yue liang zi 月亮子	Moon	Fabaceae	*Caesalpinia bonduc* (L.) Roxb.	10.32	Medicinal plant	India [7], Caribbean, Mexico, and Central America [6]	*
37	Tai yang zi 太阳子	Sun	Fabaceae	*Caesalpinia major* (Medik.) Dandy & Exell	23.02	Medicinal plant	Caribbean, Mexico, and Central America [6]	***
36	Tie lian zi 铁链子	Iron lotus seed	Fabaceae	*Caesalpinia minax* Hance	3.97	Medicinal plant		*
38	Mu yao zi 木腰子	Climbing entada	Fabaceae	*Entada phaseoloide*s (L.) Merr.	7.14	Medicinal plant	India [7,23]	*
39	Mu yu guo 木鱼果	Temple block fruit	Fabaceae	*Mucuna gigantea* (Willd.) DC.	27.78	Landscape plant	Caribbean, Mexico, and Central America [6]	*
40	Jin ling zi 金铃子	Golden jingle bell	Meliaceae	*Melia azedarach* L.	1.59	Medicinal, timber plant	India [7,24]	*
41	Jin zhong 金钟	Golden bell	Myrtaceae	*Eucalyptus exserta* F.Muell.	7.14	Medicinal, timber, oil-bearing plant		**
42	Di xue lian hua 滴血莲花	Bleeding lotus	Pandanaceae	*Pandanus tectorius* Parkinson ex Du Roi	3.17	Medicinal, oil-bearing plant		*
43	Feng yan 凤眼	Phoenix eye	Rhamnaceae	*Ziziphus abyssinica* Hochst. ex A. Rich.	57.94	-	India, Nepal [2]	***
44	Long yan 龙眼	Dragon eye	Rhamnaceae	*Ziziphus abyssinica* Hochst. ex A. Rich.	26.19	-	India, Nepal [2]	***
45	Qi lin yan 麒麟眼	Kylin eye	Rhamnaceae	*Ziziphus abyssinica* Hochst. ex A. Rich.	17.46	-	India, Nepal [2]	***
46	Lian hua 莲花	Lotus	Rhamnaceae	*Ziziphus jujuba* Miller	12.70	Medicinal, edible, timber plant		*
47	Xiao feng yan 小 凤眼	Small phoenix eye	Rhamnaceae	*Ziziphus jujuba* var. *spinosa* (Bunge) Hu ex H. F. Chow	19.84	Medicinal, edible, timber plant	India, Nepal [2]	*
48	Tao he 桃核	Peach	Rosaceae	*Amygdalus persica* L.	9.52	Medicinal, edible, timber plant		*
49	Xian tao 仙桃	Flat peach	Rosaceae	*Amygdalus persica* var. *compressa* (Loudon) T.T. Yu & L.T. Lu	0.79	Medicinal, edible, timber plant		*
50	Gui jian chou 鬼见愁	Sorrowful to a ghost	Sapindaceae	*Sapindus saponaria* L.	13.49	Medicinal, landscape plant	United States and Mexico [6]	*
51	Tian tai 天台	Tiantai	Tiliaceae	*Tilia miqueliana* Maxim.	7.14	Medicinal, landscape plant		*
52	Niu tou ma mian 牛头马面	Ox-head and horse-face	Trapaceae	*Trapa natans* L.	3.97	Medicinal, edible plant		*

(Ranked by family names alphabetically, followed by generic and species names).

Distribution: *Native to China; **Cultivated in China only; ***Not occurring in China.

Three species of Bodhi bead plants do not occur in China: *Phytelephas macrocarpa*, *Caesalpinia major*, and *Ziziphus abyssinica*. Thirty-one species are native to China, with 19 species mainly distributed in tropical and subtropical areas, and the other species widely distributed throughout China. Twelve species have only cultivation types, and most of these species have been domesticated in southwestern and southern China.

### Traditional cultural value of Bodhi bead plants

#### ***The most popular Bodhi beads and their potential cultural significance***

**Figure 1:**
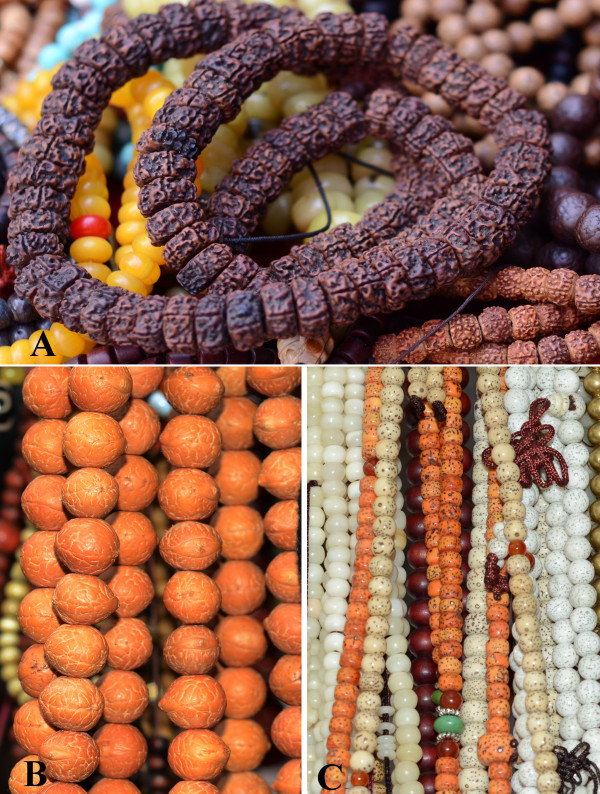
**Bodhi beads in Barkhor Street market, Lhasa. A**: ‘King Kong’; **B**: ‘Phoenix eye’; **C**: ‘Moon and star’.

The occurrence frequencies of four types of Bodhi beads reached at least 50%, including ‘King Kong’, ‘Moon and star’, ‘Bodhi root’, and ‘Phoenix eye’ (Figure 1). These were the most popular Bodhi beads in the markets.

The most common Bodhi bead, ‘King Kong’, is made from the fruit of *Elaeocarpus angustifolius*. ‘Two-furrowed King Kong’ is made from the fruit of *Elaeocarpus hainanensis. Elaeocarpus* plants are distributed in Hainan, Yunnan, Guangxi, Tibet of China, and other parts of Asia, including Nepal, Bhutan, India, Indonesia, and the lowlands of the Himalayas [27]. ‘King Kong’ may have been the earliest form of prayer bead in India [28], named ‘Rudraksha’ in the local language, meaning ‘eye of Shiva’ [27]. The furrows of the hard and rugulose endocarp of fruits are important to the rudraksha. Different numbers of furrows represent different meanings. The rough surface symbolizes the austere life expected of worshippers [28]. Five furrows and up to 14 furrows are usual, while others are very rare [2]. Normally, a string of prayer beads is made with 32, 108, or 112 beads of similar size and the same number of furrowed rudraksha seeds. The standard ‘King Kong’ string has 108 Bodhi beads, and this string of prayer beads is widely used by Tibetan Buddhists [29].

‘Moon and stars’ is a very popular and traditional Bodhi bead in Chinese Buddhism and is the hard and dense seed of *Daemonorops jenkinsiana*. The ‘Moon and stars’ name reflects the small holes (moon) and tiny black dots (stars) covering the seed’s surface. This species is mainly distributed in the south of China, India, Nepal, Bhutan and Bangladesh. There was no record of the history of this type of Bodhi bead, but we found many old ‘Moon and stars’ beads in Tibet; we therefore speculated that Tibetan Buddhism was influenced by mainland China’s Buddhism.

Seeds of *Corypha umbraculifera* are used to make ‘Bodhi root’ beads. This species is native to India and Sri Lanka. In India, it is also called ‘vegetable ivory’ and is a traditional tool for carving Buddhist Sutras, such as the famous ‘tale palm’ or ‘tad-patri’ because the leaves are flexible and soft when dry [30-32]. Because of early Indian Buddhist influences [33], *C. umbraculifera* was cultivated in temple gardens in Xishuangbanna of Yunnan Province in China, and over 50,000 volumes of Buddhist Sutras carved on leaves were protected there [34]. In addition, ancient books and letters of Southeast Asian countries were written on its leaves [35]. Therefore, *C. umbraculifera* is regarded as one of the iconic Buddhist plants in Asia.

‘Phoenix eye’ and ‘Small phoenix eye’ are made from the fruits of *Ziziphus abyssinica* and *Ziziphus jujuba* var. *spinosa*, respectively. The name ‘Phoenix eye’ refers to the eye-like shape that appears on the hard endocarp. *Z. abyssinica* is native to India and is not cultivated in China. Both were used to make adornments in India [7,36]. *Ziziphus* has been mentioned more than one time in the Ramayana and the Mahabharata [37]. The tree and fruit of this genus have great significance in Indian traditional culture. Jujube trees are one of the few sacred trees of the Sikhs in India. The species of jujube tree named Christ’s Thorn Jujube (*Z. spina-christi*) is the only tree that could be regarded as a holy tree in Islam [38]. Species of *Ziziphus* are also held sacred by many other religious persons, such as some Druze, Muslims, and Christians.

### Traditional playing nuts in China

Chinese olives (*Canarium* spp.) have a long history as accessories in China. Drupes of Chinese black olives (*Canarium pimela*) are the main material for carving and playing and are produced mainly in the Chinese provinces of Guangdong and Fujian. ‘Olive-stone carving’ prospered in the Ming to Qing (1368–1911) dynasties, although it might have originated earlier. The most classical ‘olive-stone carving’ is the ‘Olive nut boat.’ Playing with walnuts is a traditional Chinese practice with its origin in traditional Chinese medicine therapies: the Chinese consider the bumps and sharp edges of a walnut to promote blood circulation when playing with walnuts in one’s hands [39]. Each *Juglans* species in China can be used for playing with walnuts, including *Juglans regia*.

### Similar uses of Bodhi bead plants in different countries

Eighteen species presented in the present paper were also used for making ornaments in other countries. Seeds of 12 species were strung for necklaces and strings of prayer beads in India. In addition to *E. angustifolius*, *C. umbraculifera*, *Z. abyssinica*, and *Z. jujuba*, plants used as adornment in India include *Thevetia peruviana*, *Abrus precatorius*, *Areca catechu*, *Coix lacryma-jobi*, *Adenanthera pavonina*, *Caesalpinia bonduc*, *Entada phaseoloides*, and *Melia azedarach*. Ten species were used in the American continent, as well. Most of these species were used as a simple necklace without religious meaning. Seeds of *A. precatorius* were widely used because of their strikingly beautiful appearance and amazing durability. The world-famous ‘plant elephant’ is the seed of *Phytelephas macrocarpa* and is a type of precious Bodhi bead in China named ‘Ivory nut.’

### Multiple uses of Bodhi bead plants in China

In addition to making Bodhi beads, all Bodhi bead plants in China have other uses. Thirty-six species (76.6%) are also used as medicine. The seeds or fruits of most species are the main medicinal parts. Some species can be used to treat heart disease, dyspepsia, hypertension, rheumatism, and other ailments. Except for *Mucuna gigantea*, all seeds of the legume family used to make Bodhi beads have medicinal functions. For example, seeds of *Abrus precatorius*, *Acacia confusa*, and *Adenanthera pavonina* can be used to treat skin diseases. Seeds of *Afzelia xylocarpa* have an anti-inflammatory function, while seeds of *Caesalpinia bonduc* or *C. minax* can be used to treat rheumatism [40]. Although there was no record about whether *C. major* was cultivated in China, it has been recorded as a traditional Chinese medicine [41]. Twenty species (42.6%) of Bodhi bead plants are also used as landscape plants or for aesthetic purposes, especially the species in Arecaceae. Seeds of these plants are the main sources of materials for making Bodhi beads. Most of the Arecaceae species are cultivars introduced into China and were mainly cultivated as ornamental plants in parks, scenic spots, or roadsides. Five species in Arecaceae are important fiber plants (10.6%). The stem of *Daemonorops jenkinsiana* can be used for furniture-making and is an important source of rattan material in South China [12]. Leaves of *Corypha umbraculifera* can remain flexible for a long period and can be used for making fans, mats, umbrellas, baskets, thatching, roofing, and so on [32]. Sixteen species of Bodhi bead plants (34.0%) are edible. The main edible parts are their seeds or fruits, such as *Dracontomelon macrocarpum* and *Ziziphus jujuba* var. *spinosa*. The central soft part of the stem of *C. umbraculifera* is a rich source of starch that can be made for sago. The most valuable commercial Bodhi bead plants are four oil-bearing species and nine timber species. *Elaeis guineensis* is called oil palm and is the main oil-bearing crop in tropical areas. The oil content of its fruit is higher than 50%, and this species has the highest productivity of any oil-bearing plant in the world [42].

## Conclusion

As one of the most popular Buddhist supplies, Bodhi beads are being paid more attention and becoming the target of many collectors. However, the sources of Bodhi beads have been ignored.

The purpose of the current study was to give an account of Bodhi bead plants and the reasons for the widespread use of Bodhi beads in China. Seeds of 47 species (2 varieties) were recorded to be used for making 52 types of Bodhi bead. Some of these seeds were also used in other countries as necklaces or strings of prayer beads. They might be the oldest materials used for ornaments. Twelve of them were traditional Indian beads, especially rudraksha seeds. Together with other items, they were the earliest prayer beads in Indian Buddhism. We also found that all of the most popular Bodhi bead plants had a close historical relationship with religion. Bodhi bead plants have multiple uses in China. Besides aesthetic functions, they can be used as food, medicine, timber, and plant oil. These seeds and other parts of plants played a prominent role in the lives of humans for thousands of years. Bodhi beads might be the byproducts of plants, and use of seeds for Bodhi beads might have increased the conservation value of these plants and helped biodiversity conservation in China. The evidence from this study suggests that Buddhism culture combined with Chinese collection culture resulted in modern Bodhi bead culture and that commercial interest in Bodhi beads promotes the spread of Bodhi bead culture.

## Competing interests

The authors declare that they have no competing interests.

## Authors’ contribution

FL, JL, JZ, and CL participated in the field survey and conceived of the study. FL, CL, and BL identified all Bodhi bead plants. FL coded all the data and wrote the first draft of the manuscript. All authors contributed to the interpretation of results and contributed to the final manuscript. All authors read and approved the final manuscript.

## Authors’ information

Chunlin Long is a professor at the College of Life and Environmental Sciences, Minzu University of China, Beijing, and research professor at the Kunming Institute of Botany, Chinese Academy of Sciences, Kunming. His research focuses on ethnobotany and ethnomedicine, biodiversity, and plant genetic resources. Feifei Li is a postdoctoral research fellow at the College of Life and Environmental Sciences, Minzu University of China, Beijing. Jianqin Li is an associate professor at the Faculty of Forestry, Southwest Forestry University, Kunming, and Ph.D. candidate at the College of Life and Environmental Sciences, Minzu University of China, Beijing. Bo Liu is a lecturer at the College of Life and Environmental Sciences, Minzu University of China, Beijing. Jingxian Zhuo is a master degree student at the College of Agronomy and Biotechnology, Yunnan Agricultural University, and the Kunming Institute of Botany, Chinese Academy of Sciences, Kunming.

## References

[B1] Carey M Beads and beadwork of East and South Africa 1986 Aylesbury: Shire Publications

[B2] Majupuria TC Joshi DP Religious & useful plants of Nepal & India 1997 India: Gupta Lashkar

[B3] Li S Luo X Compendium of materia medica: bencao gangmu 2003 Beijing: Foreign Languages Press

[B4] Wu Z Flora of Yunnan 1977 Beijing: Science Press

[B5] Armstrong WP Beautiful botanicals: seeds for jewelry Ornament 1991 15 66 69

[B6] Armstrong WP Jewels of the tropics Terra 1992 30 26 33

[B7] Francis P Plants as human adornment in India Econ Bot 1984 38 194 209 10.1007/BF02858832

[B8] Dowman K The power-places of Central Tibet: the pilgrim’s guide 1988 London: Routledge & Kegan Paul

[B9] Hooker JD The Flora of British India 1890 London: Reeve & Company

[B10] Press JR Shrestha KK Sutton DA Annotated checklist of the flowering plants of Nepal 2000 London: Natural History Museum Publications

[B11] Flora of Pakistan [http://www.tropicos.org/Project/Pakistan]

[B12] Wu Z Shehbaz IAA Bartholomew B Flora of China 1994 Beijing, St. Louis: Science Press, Missouri Botanical Garden Press

[B13] Ortiz de Montellano B Aztec medicine, health, and nutrition 1990 New Jersey: Rutgers University Press

[B14] Balfour E Asylum, Scottish, and Foster, Madras Cyclopedia of India. Volume 2 2012 Charleston: Nabu Press

[B15] Armstrong WP Vegetable ivory: saving elephants & South American rain forests Zoonooz 1991 64 17 19

[B16] Elevitch CR Manner HI Elevitch CR Aleurites moluccana (kukui) Traditional trees of Pacific Islands: their culture, environment and use Permanent Agriculture Resource, Holualoa 2006 Holualoa, Hawaii: Permanent Agriculture Resources (PAR) 41 56

[B17] Armstrong WP Job’s tears Ornament 1994 18 104 105

[B18] Watt G A dictionary of the economic products of India 1893 India: Printed by the Superintendent of Government Printing

[B19] Roy S The Bihors 1925 Ranchi: Mission Press, Ranchi

[B20] Vd G Primitive India 1954 London: Harrap

[B21] Burkill I A dictionary of the economic plants of the Malay Peninsula Crown Agents for the Colonies, London. Volume 2 1935 Kuala Lumpur: The Ministry of Agriculture and Cooperatives

[B22] Balée WL Footprints of the forest: Ka’apor ethnobotany-the historical ecology of plant utilization by an amazonian people 1999 New York: Columbia University Press

[B23] Mehra K Kanodia K Srivastava R Folk uses of plants for adornment in India Econ Bot 1975 29 39 46 10.1007/BF02861254

[B24] Dastur JF Useful plants of India and Pakistan 1963 Bombay: DB Taraporawala Sons and Company Pvt. Limited

[B25] Commission ISS IUCN Red List of threatened species World Conservation Union, Gland, Switzerland (www redlist org) 2000

[B26] Wang S China species red list 2004 Beijing: Higher Education Press

[B27] Lee DW The biology of rudraksha Curr Sci 1998 75 26 30

[B28] Dubin LS The history of beads from 30,000 BC to the present 1987 London: Harry N. Abrams. Inc.

[B29] Blackman WS The rosary in magic and religion Folklore 1918 29 255 280 10.1080/0015587X.1918.9719067

[B30] Kulkarni A Mulani R Indigenous palms of India Curr Sci 2004 86 1598 1603

[B31] Bhowmik S Conservation of palm-leaf manuscripts Baroda Museum and Picture Gallery, Bull 1965 19 59 65

[B32] Kumar DU Sreekumar G Athvankar U Traditional writing system in southern India—palm leaf manuscripts Design Thoughts 2009 7

[B33] Wong KC Chinese culture and leadership Int J Leadersh Educ 2001 4 309 319 10.1080/13603120110077990

[B34] Liu H Xu Z Xu Y Wang J Practice of conserving plant diversity through traditional beliefs: a case study in Xishuangbanna, southwest China Biodivers Conserv 2002 11 705 713 10.1023/A:1015532230442

[B35] Thammincha S Suksard S Tanasombat M Pisuttipiched S Riddiboot K Historical background of paper production and utilization in Thailand 1996 Thailand: Thai National AGRIS Centre

[B36] Griffiths WG Guha B The Kol tribe of central India 1946 Kolkata: Royal Asiatic Society of Bengal

[B37] Gupta SM Plant myths and traditions in India 2001 Calcutta: Munshiram Manoharlal

[B38] Dafni A On the present-day veneration of sacred trees in the holy land Folklore-Electronic J Folklore 2011 48 7 30

[B39] Li H Li W Ma Y Ma Y Study on the value of walnut culture Forest Inventory and Planning 2011 3 023

[B40] Assogbadjo AE Glèlè Kakaï R Adjallala FH Azihou AF Vodouhê GF Kyndt T Codjia JTC Ethnic differences in use value and use patterns of the threatened multipurpose scrambling shrub (Caesalpinia bonduc L.) in Benin J Med Plants Res 2011 5 1549 1557

[B41] Zhou J Xie G Yan X Encyclopedia of Traditional Chinese Medicines: Molecular Structures, Pharmacological Activities, Natural Sources and Applications. Indexes 2011 Heidelberg: Springer

[B42] Xiong H Li R Li X Fan H Ma Z Investigation, analysis and the advice of palm industry in china Chin Agric Sci Bull 2009 24 023

